# Recent advances in understanding and treating immunoglobulin light chain amyloidosis

**DOI:** 10.12688/f1000research.15353.1

**Published:** 2018-08-29

**Authors:** Talha Badar, Anita D'Souza, Parameswaran Hari

**Affiliations:** 1 Division of Hematology and Oncology, Medical College of Wisconsin, Milwaukee, Wisconsin, USA

**Keywords:** AL amyloidosis, cardiac amyloidosis, dFLC, autologous transplant for AL amyloidosis

## Abstract

Immunoglobulin (Ig) light chain (AL) amyloidosis is a clonal plasma cell disorder characterized by misfolded Ig light chain deposition in vital organs of the body, resulting in proteotoxicity and organ dysfunction. Owing to its diverse clinical presentations and a tendency to mimic common medical conditions, AL amyloidosis is often diagnosed late and results in dismal outcomes. Early referral to a specialized center with expertise in management of AL amyloidosis is always recommended. The availability of sensitive biomarkers and novel therapies is reforming our approach to how we manage AL amyloidosis. Treatment for patients with AL amyloidosis should be risk-adapted and customized on the basis of individual patient characteristics. In the future, approaches directed at amyloid fibril clearance in combination with agents that target plasma cells will be needed both to eradicate the malignant clone and to establish organ responses.

## Introduction

Amyloidosis is a plasma cell disorder characterized by the extracellular deposition of pathologic, insoluble fibrillary protein in various organs of the body, leading to organ dysfunction ^1^. Amyloidosis can manifest as a localized disease or can present as systemic disease with multiple organ involvement. Localized deposits of amyloid can occur anywhere in the body and are usually light chain (LC). Common sites of localized amyloid LC (AL) amyloidosis include the orbit, pharyngeo-laryngeal region, tracheobronchial tree, pulmonary parenchyma, and urinary bladder. Localized amyloidosis has excellent prognosis, usually does not affect life expectancy, and rarely evolves to systemic disease ^2^. Systemic amyloidosis is more common than localized amyloidosis and can have serious implications. The common proteins involved in systemic amyloidosis and organ dysfunction are immunoglobulin (Ig) LCs (AL), Ig heavy chain (AH), transthyretin (ATTR), serum amyloid A (AA), apolipoprotein A-I (AApoAI), β2-microglobulin, and leukocyte chemotactic factor-2 (ALECT2) ^3^. Types of systemic amyloidosis are summarized in Table 1. The most common among them in the US and Europe is AL amyloidosis, which is caused by deposition of misfolded LCs produced by a neoplastic plasma clone. In this review, we will discuss the pathophysiology, recent progress in diagnostic modalities, newer therapeutic strategies, including immunotherapy, and the role of transplant in systemic AL amyloidosis.

**Table 1.** Types of systemic amyloidosis.

Disease	Organ involvement	Amyloid protein	Incidence
**AL amyloidosis (AL)**	Kidney, heart, liver, GI, soft tissue, PNS, and ANS	Ig light chain	9–14 per million person-years
AH amyloidosis (AH)	Mainly kidney involvement	Ig heavy chain	Rare, incidence not reported
Transthyretin amyloidosis (ATTR)	Heart, PNS, kidney, and eye	Transthyretin	70–79 years; 1.6% in AA and 0.1% in Caucasians >90 years; 8.2% in AA and 2.7% in Caucasians
Familial amyloidosis (AF)	Renal, PNS, GI, and eye	Transthyretin, apolipoprotein AI, and cystatin C	Rare, incidence not reported
Apolipoprotein AL amyloidosis (AApoAI)	Kidney, liver, PNS, heart, and skin	Apolipoprotein AL	Rare, incidence not reported
AA amyloidosis (AA)	Kidney, liver, GI, ANS, and thyroid	Serum amyloid A (SAA)	0.5–0.86%
Dialysis-related amyloidosis (Aβ2M)	Osteoarticular tissue; infrequently involves GI, blood vessels, and heart	β2-microglobulin	21% patients on HD <2 years 50% patients on HD 4–7 years 90% patients on HD 7–13 years

AA, African-Americans; ANS, autonomic nervous system; GI, gastrointestinal tract; HD, hemodialysis; Ig, immunoglobulin; PNS, peripheral nervous system.

## Pathophysiology

### Molecular events

The molecular events that take place in AL amyloidosis start with a clonal expansion of differentiated plasma cells leading to the production of amyloidogenic LCs characterized by instability and improper aggregation ^4^. These LCs and their fragments interact with extracellular matrix and glycosaminoglycans (GAGs) forming oligomers ^5, 6^. Serum amyloid proteins (SAPs) present in amyloid deposits prevent reabsorption of these amyloid fibrils/oligomers ^7^. Replacement of normal tissue architecture with pathologic amyloid deposits leads to organ dysfunction.

### Organ toxicity and tropism in amyloid

The exact mechanism of amyloid deposition in various organs of the body and consequent toxicity is not completely understood. Several mechanisms of amyloid-related proteotoxicity leading to organ dysfunction have been reported in the literature. Migrino *et al*., in *ex vivo* experiments, demonstrated that AL-related myocardial dysfunction is a result of increased oxidative stress, leading to decreased nitrous oxide availability and apoptotic injury of cardiac endothelium ^8^. Colleagues from Boston University demonstrated that impaired lysosomal function is the major cause of defective autophagy and LC-induced proteotoxicity ^9^. A recent study by Marin-Argany *et al.* ^10^ suggested that LC fragments of amyloid are toxic even at low concentrations. They can aggregate and recruit soluble proteins, resulting in elongation of amyloid fibrils and cellular toxicity. Shi *et al*. proposed that the activation of p38 mitogen-activated protein kinase (MAPK) is one of the molecular mechanisms responsible for cardiotoxicity through an increase in cellular oxidative stress and apoptosis ^11^. This mechanism is independent of MAPK but rather dependent on transforming growth factor-beta–activated protein kinase-1 binding protein-1–dependent autophosphorylation. It is important to note that p38 MAPK signaling is the same pathway that mediates type B natriuretic peptide (BNP) transcription, supporting an association between LC cardiotoxic effects with induced MAPK signaling and elevated BNP levels. For renal amyloid, researchers have conducted *in vitro* and *in vivo* studies demonstrating that the interaction of mesangial cells with internalized LC causes the formation of amyloid fibrils, which then causes extracellular matrix proteotoxicity through lysosomes ^12^.

It has been hypothesized that AL amyloid manifests organ tropism which may be a function of LC variable region gene polymorphisms ^13, 14^. Studies have shown that LC variable region gene subtypes predispose to specific organ involvement in AL. Perfetti *et al*. ^13^ conducted a study in which biopsy specimens of patients with AL amyloidosis were examined by liquid chromatography mass spectrometry. The study demonstrated that AL amyloidosis patients with LV2-14 mutation were more likely to have peripheral nerve involvement, LV1-44 mutated were more likely to have cardiac involvement and KV1-33 mutated were associated with liver involvement.

### Amyloidogenic clone

AL amyloidosis is caused by a clonal expansion of differentiated plasma cells, which produces misfolded LCs that have the propensity to deposit in the vital organs of the body, commonly heart, kidney, liver, and nervous system ^15^. The presence of greater than 10% clonal plasma cells in the bone marrow is associated with inferior outcomes regardless of the presence or absence of myeloma like end-organ damage caused by the plasma cell clone ^16^. Next-generation sequencing data suggest that the mutational pattern of AL amyloidosis is intermediate between monoclonal gammopathy of undetermined significance (MGUS) and multiple myeloma. The t(11;14) and hyperdiploidy are the most commonly observed chromosomal abnormalities in amyloidogenic plasma cells; these chromosomal abnormalities are associated with the size of the plasma cell clone and relative resistance to proteosome inhibitor-based therapy ^17^. Moreover, gain of 1q21 is associated with poor response to conventional melphalan-based chemotherapy that can be overcome with proteasome inhibitor-based therapy ^18^. Hammons *et al*. conducted a retrospective review of 107 patients with AL and evaluated the prognostic impact of chromosomal abnormalities detected by fluorescent *in situ* hybridization (FISH) on diagnostic bone marrows. The study did not show any correlation between AL stage or survival at one year and chromosomal abnormalities detected by FISH, including t(4;14), 1q21, del 17p, and hypodiploidy ^19^.

## Recent advances in diagnostic modalities

### Biomarkers

Delayed diagnosis occurs in about 40% of patients with AL amyloidosis and among them 25% of patients will present with advanced cardiac disease having dismal outcome ^20^. Increased awareness about the disease and improved diagnostic modalities are needed to diagnose early and manage patients efficiently.

Over the last decade, the availability of biomarkers such as N-terminal pro-brain natriuretic peptide (NT-proBNP) has improved diagnostic accuracy and risk stratification ^21^. In published literature, NT-proBNP in cardiac amyloid and albuminuria in renal amyloid have shown high diagnostic accuracy ^22^. This has encouraged clinicians to adopt biomarker-based screening for AL amyloidosis in patients with monoclonal gammopathy of undetermined significance.

Moreover, quantification and monitoring of Ig free LC (FLC) assay have been validated for diagnosis and risk stratification and to assess response to treatment ^23^. Lately, sensitive mass spectrometry–based technologies have been developed for monoclonal FLC detection and quantification, which have improved test sensitivity ^24^.

Timing of treatment during relapse in AL has been controversial because of the time delay between hematological and subsequent organ progression. Palladini *et al*. recently evaluated clinical outcome of patients with relapsed AL amyloidosis who had initially responded to non-transplant therapies ^25^. They identified patients with probability for high risk of progression on the basis of difference between involved and uninvolved FLC (dFLC). Patients with a dFLC of more than 20 mg/dL or a 20% increase in dFLC from baseline at relapse or more than 50% increase in dFLC from nadir were defined as “high-risk dFLC progression” and these patients had inferior outcomes despite relatively small increases in FLC ^25^. The study suggests that patients with high-risk dFLC receive salvage therapies earlier before clinical evidence of organ progression.

### Imaging

For several decades, echocardiography has been the diagnostic imaging modality of choice for detecting cardiac amyloid. Recently, advanced cardiac magnetic resonance imaging using T1 mapping and extracellular volume measurements has been adopted for diagnosis of cardiac amyloid with good specificity and capability to provide desired anatomical details and prognostic information ^26^. Additionally, recognizing distinct myocardial strain patterns and its association with cardiac amyloidosis has further enhanced the utility of cardiac imaging in amyloid ^27^. Tracers used for imaging β-amyloid protein in the brain for Alzheimer’s disease— ^11^C-labeled Pittsburgh compound B ( ^11^C-PIB), ^18^F-florbetapir, and ^18^F-florbetaben—have very high sensitivity for amyloid and have been used for imaging cardiac AL amyloidosis ^4, 28^. The bone-seeking radionucleotide tracers ^99m^Tc-3,3-diphosphono-1,2 propanodicarboxylic acid, ^99m^Tc-hydroxymethylene diphosphonate, and ^99m^Tc-pyrophosphate have shown high sensitivity for cardiac ATTR deposits and can be used to differentiate AL amyloidosis ^29^.

Few novel strategies have been proposed in the past to efficiently diagnose systemic amyloidosis but none of them has been implemented in clinics yet. Hawkins *et al*. proposed radionuclide imaging using serum amyloid P (SAP) with the capability of evaluating the kinetics of amyloid deposition and regression of amyloid precursors after therapy ^30^. One drawback of this test is that it cannot detect cardiac amyloid. Another innovative strategy is to use serine protease inhibitor (aprotinin) labeled with technetium 99 ( ^99m^Tc) that potentially detects early amyloid deposition in vital organs of the body ^28^. These tests require further study and validation before being implemented in clinical practice.

## Amyloid typing

Once amyloid is identified, typing should be carried out for further characterization. Historically, immunohistochemistry was used for amyloid typing but the test has low sensitivity and specificity. Since then, electron and immuno-electron microscopy has been shown to correctly identify and characterize patients with amyloidosis ^31^. Similarly, direct immunofluorescence microscopy is another method for amyloid typing, commonly performed on kidney biopsies. Immunofluorescence using fluorescein isothiocyanate (FITC)-labeled antibodies to IgA, IgM, IgG, κ, and λ LC are used for amyloid typing. Vrana *et al*. reported a more sensitive method of amyloid typing by combining laser microdissection and mass spectrometry–based proteomic analysis ^32^. The test was validated in 41 cardiac amyloid patients with 100% sensitivity and specificity. Once the Ig component involved in amyloidogenesis is identified, further therapy can be implemented.

## Clinical presentation

Clinical presentation is variable depending on the organ involved. AL amyloidosis can involve any organ in the body except the brain:heart (82%), kidney (68%), soft tissue (17%), liver (14%), peripheral/autonomic nervous system (12%), and gastrointestinal tract (8%) ^4^. Among them, cardiac involvement is the main determinant of early mortality ^33^. Early diagnosis is vitally important before the development of advance cardiomyopathy to improve outcomes. There are certain “red flag” signs of AL that all physicians should be aware of in order to make a prompt diagnosis; these include soft tissue amyloid infiltrates (that is, macroglossia and bilateral carpal tunnel syndrome), nephrotic syndrome, autonomic dysfunction (that is, orthostatic hypotension, nocturnal diarrhea, and erectile dysfunction), bleeding (that is, cutaneous and periorbital ecchymosis), and genetic predisposition (that is, family and ethnicity).

## Treatment

The outcome of AL amyloidosis with advanced disease has improved in the most recent decade with the incorporation of novel drugs such as proteasome inhibitors and consolidation therapy with autologous hematopoietic cell transplant (autoHCT). A recent report of 1,551 patients with AL amyloidosis revealed that about one third of the patients are achieving a median survival of more than 10 years ^34^. In recent years, early mortality appears to have improved from 40% at 6 months ^35^ to 24% at 6 months ^34^.

### Novel therapies

Before the advent of novel therapies, limited options existed for patients with AL amyloidosis. For several years, melphalan-dexamethasone (MDex) was the standard-of-care upfront treatment for AL amyloidosis ^36^. The introduction of bortezomib has been a revolution in producing rapid hematologic responses in patients with AL amyloidosis. In a matched control study of AL amyloidosis, complete response (CR) was observed in 42% of patients who received a bortezomib, melphalan, and dexamethasone (BMDex) combination compared with 19% with MDex alone ^37^. In another study, data of 230 patients with AL amyloidosis were retrospectively evaluated for response to an upfront cyclophosphamide, bortezomib, and dexamethasone (CyBorD) combination regimen. The overall hematological response rate observed was 60%; among them, 23% achieved CR ^38^.

A lenalidomide, melphalan, and dexamethasone (LMDex) combination has been tested in transplant-ineligible, treatment-naïve patients and demonstrated response rates of 50% (n = 25/50) compared with 24% (n = 12/49) in historical matched patients who received MDex only. Higher response rates with LMDex regimen were translated into improved event-free survival and overall survival (OS) ^39^. Similarly, an upfront lenalidomide, cyclophosphamide, and dexamethasone combination was evaluated in a single-arm, phase II study in patients with AL amyloidosis ^40^. The overall response rate (ORR) was 60%, but none of the patients achieved CR. Hematological toxicity was the major adverse event noted.

Immunomodulatory agents (IMiDs), including thalidomide ^41^, lenalidomide ^42^, and pomalidomide ^43^, have shown significant activity in patients with relapsed refractory AL amyloidosis with the hypothesis that these drugs can overcome the resistance to alkylating agent therapy. In the relapsed refractory population, treatment with IMiDs demonstrated ORR in the range of 50 to 70%, although a smaller proportion of patients achieved CR ^42, 43^. However, IMiD-based regimens should be used with caution in patients with proteinuria. Moreover, these drugs can be associated with significant hematologic toxicities, fluid retention, and increase in cardiac biomarkers in AL amyloidosis and hence these patients warrant active surveillance while on therapy. Table 2 summarizes salvage chemotherapy regimens in patients with relapsed refractory AL amyloidosis.

**Table 2.** Outcome of relapsed refractory amyloid light-chain amyloidosis with salvage treatment regimens.

Study	Treatment	Patients, number	Hematological response percentage (CR percentage)	Organ response	Progression- free survival	Overall survival
Phase III ^48^	HDM → autoHCT versus MDex	100 (50 in each group)	72 versus 74	45% versus 39%	32 versus 32.5 months	22.2 versus 56.9 months
Retrospective ^49^	Bendamustine + prednisone	36	47 (3)	12%	NR	65% alive at 3 years
Phase II ^43^	Pomalidomide and dexamethasone	28	68 (29)	Renal response 2/12 (17%)	16 months	26 months
Phase I/II ^50^	Ixazomib	27	52(4)	56% (renal 45%, cardiac 45%)	15 months	86% at 1 year
Phase I ^51^	Carfilzomib	24	63 (13)	21%	20 months	NR

autoHCT, autologous hematopoietic cell transplant; CR, complete response; HDM, high-dose melphalan; MDex, melphalan + dexamethasone; NR, not reached.

### Immunotherapy

Daratumumab is a human IgG1κ monoclonal antibody that targets CD38 surface antigen on plasma cells and is now an important component of multiple myeloma therapy. Daratumumab has recently been evaluated in patients with AL amyloidosis. In one retrospective analysis of 25 relapsed refractory patients, it showed a remarkable ORR of 76% and a CR rate of 36% ^44^. Consequently, several prospective trials are evaluating efficacy of daratumumab in AL amyloidosis, including a phase 3, CyBorD plus daratumumab versus CyBorD alone as upfront therapy (ClinicalTrials.gov Identifier NCT03201965), daratumumab as a single agent in relapsed refractory AL amyloidosis (ClinicalTrials.gov Identifier NCT02816476), and daratumumab, ixazomib, and dexamethasone in previously treated AL amyloidosis (ClinicalTrials.gov Identifier NCT03283917).

### Fibril-directed therapies

Doxycycline is a bacteriostatic antibiotic that binds to 30S and 50S ribosomal subunits, inhibiting protein synthesis. It has been shown to interfere with amyloid fibril formation in pre-clinical studies ^45^. Subsequently, in a retrospective analysis of 30 cardiac amyloid patients and comparison with matched controls, the addition of doxycycline to standard chemotherapy suggested improved survival compared with historic matched controls ^46^. Another agent discovered to have anti-fibril properties is epigallocatechin gallate (ECGC), which is one of the main polyphenols in green tea. An interesting observation was made in a retrospective analysis of 59 cardiac amyloid patients who consumed green tea regularly. Among them, 11 (19%) patients had reduction in interventricular wall thickness by at least 2 mm ^47^. Prospective studies are ongoing to study the efficacy of ECGC in patients with cardiac amyloid (ClinicalTrials.gov Identifier NCT02015312). Table 3 summarizes innovative approaches in the management of patients with AL amyloidosis.

**Table 3.** Novel therapies for light-chain amyloidosis.

Study	Treatment	Patients, number	Response	Adverse events
Retrospective ^44^	Daratumumab	25	HR: ORR 76%, CR 36%	Infusion reaction GI-II
Retrospective ^60^	Doxycycline ^a^	30	HR 56% versus 33%	Photosensitive rash 3
Phase I/II ^52^	NEOD001 ^b^	27	Cardiac 43%, renal 60%	None
Case control ^47^	Epigallocatechin-3-gallate ^c^	59	Cardiac 19%	None
Phase I/Ib ^61^	CAEL-101	24	Cardiac 67%, renal 50%	Diarrhea and rash GI-II

^a^Given adjuvant to chemotherapy compared with matched control. ^b^Monoclonal antibody targeting misfolded protein. ^c^Given in addition to standard therapy. CR, complete response; G, grade; HR, hematological response; ORR, overall response rate.

A novel approach is to use monoclonal antibody to target amyloid fibrils. The monoclonal antibody, NEOD001, directed against an LC cryptic epitope on amyloid fibrils was developed and evaluated in a phase I/II study ^52^. Among the 27 patients who had more than partial response with previous therapy, NEOD001 showed cardiac and renal response rates of 57% and 60%, respectively. Subsequently, phase IIb and III studies of NEOD001 were conducted but found to be ineffective. The drug has since been discontinued in AL ^53^.

A second novel chimeric fibril-reactive monoclonal antibody, II-IF4 (CAEL-101), has also been evaluated in a phase 1/1b study in patients with relapsed refractory AL amyloidosis. The study demonstrated overall organ response of 63% (14/24), 67% (8/12) cardiac response, and 50% (5/10) renal response ^54^. The median times to response were 21 and 28 days in cardiac and renal amyloid, respectively. For further evaluation of the effectiveness of CAEL-101, phase IIb/III studies are planned to be conducted soon. Other fibril-directed strategies for amyloidosis include dezamizumab, an anti-SAP antibody, combined with miridesap; a small-molecule CPHPC which depletes circulating SAP, showed activity in a phase I clinical trial in effectively removing amyloid deposits from liver, kidney, and other organs ^55^. A phase II study evaluating the efficacy of anti-SAP monoclonal antibody in cardiac amyloid is ongoing in Europe and the US (ClinicalTrials.gov Identifier NCT03044353).

## Autologous hematopoietic cell transplant

Compared with multiple myeloma, autoHCT in AL is associated with higher morbidity and mortality. A randomized phase III trial comparing autoHCT with oral melphalan/dexamethasone in AL amyloidosis was completed in 2007 ^48^. The study did not show a survival benefit with autoHCT and was associated with high transplant-related mortality (TRM) of around 24%. Lack of benefit from autoHCT may be due to the fact that 13 out of 50 patients randomly assigned to the autoHCT arm did not receive transplant and one third of the transplanted patients received a lower dose of melphalan for conditioning. Subsequently, a large database analysis of over 1,500 AL amyloidosis patients who underwent autoHCT between 1995 and 2012 in the US and Canada showed significantly decreased day 100 post-transplant mortality from 20% (1995–2000) to 5% (2007–2012) ^56^. Estimated five-year OS had also improved from 55% (1995–2000) to 77% (2007–2012). Furthermore, transplants carried out at centers conducting more than at least four transplants per year had lower TRM. Factors associated with survival included performance score, absence of cardiac amyloid, or renal failure. Thus, among carefully selected patients, autoHCT can be used with good effect and can improve long-term outcomes. Recently, reports suggest that risk-adaptive dosing of melphalan and consolidation therapy post-transplant with novel agents such as bortezomib can decrease TRM and improve response rates in patients with AL amyloidosis ^57^.

## Supportive care in amyloid light-chain amyloidosis

Since AL amyloidosis can involve multiple organs and have a wide spectrum of clinical manifestations, good supportive care is equally important, especially during the peri-transplant and active chemotherapy phases as these treatments have inherent organ toxicities ^48, 58^. Fluid management should be dealt with cautiously as there is a risk of cardio-renal syndrome even with mild intravascular volume depletion. Studies have shown that AL amyloidosis is more prone to bleeding due to related bleeding diathesis and can have an acquired factor X deficiency; hence prophylactic anti-coagulation should be used with caution ^59^. Severe autonomic neuropathy associated with AL amyloidosis can lead to debilitating orthostatic hypotension but may be managed with alpha-1 adrenergic agonists such midodrine and other measures such as anti-gravity stockings. Patients with severe malnutrition from gastrointestinal amyloidosis may need parenteral nutrition. Thus, the management of patients with AL amyloidosis warrants a multi-disciplinary team approach with immense care toward supportive measures in addition to chemotherapy.

## Conclusions and future directions

The outcome of AL amyloidosis has improved over the last decade with more sensitive diagnostic modalities, incorporation of novel therapies, improved risk stratification for therapies, and improved supportive care. But there are still several areas of improvement for this rare and orphan disease. Significant proportions of patients are diagnosed too late, and delayed diagnosis remains a critical unmet need for patients. Increased awareness among all clinicians regarding the diverse yet unique clinical presentation of AL amyloidosis is of key importance. Incorporation of more sensitive biomarkers with novel imaging methods specific to amyloid, such as amyloid-sensitive nuclear imaging, can help in early diagnosis and more efficient management. Utilization of immunotherapy as a bridge to transplant in patients ineligible for high-dose chemotherapy can help in decreasing amyloid clone and improving patient eligibility for transplant. Newer fibril-directed therapies may further improve early mortality of patients in the first 6 to 12 months after diagnosis. In the future, the development of therapies targeting stabilization of the LC variable region, counteracting proteotoxicity caused by misfolded Ig LC, has the potential to improve outcomes of patients with AL amyloidosis.
